# Complete Genome Sequence of a Cognatishimia activa Strain Assembled from the Phycosphere of the Marine Diatom Skeletonema tropicum

**DOI:** 10.1128/mra.01164-22

**Published:** 2023-01-04

**Authors:** Kangli Guo, ShuaiShuai Xu, Yuanhao Liu, Zhuo Chen, Shengwei Hou

**Affiliations:** a Shenzhen Key Laboratory of Marine Archaea Geo-Omics, Department of Ocean Science and Engineering, Southern University of Science and Technology, Shenzhen, China; b Southern Marine Science and Engineering Guangdong Laboratory (Guangzhou), Guangzhou, China; c College of Life Science and Technology, Jinan University, Guangzhou, China; d College of Life Sciences, Shandong Normal University, Jinan, China; e State Key Laboratory for Marine Environmental Science, Institute of Marine Microbes and Ecospheres, Xiamen University, Xiamen, China; DOE Joint Genome Institute

## Abstract

Cognatishimia activa, previously known as *Thalassobius activus*, has been frequently isolated from marine environments. Here, we present the complete genome sequence of *C*. *activa* strain SOCE 004, assembled from the phycosphere of a long-term laboratory-maintained culture of the diatom Skeletonema tropicum. The complete genome is 3,211,994 bp long, with an average G+C content of 53.69%. The genome contains 3,195 genes, including 3,133 protein-coding genes, 50 tRNAs, and 3 copies each of 5S, 16S, and 23S rRNA genes.

## ANNOUNCEMENT

Bacteria inhabiting the phycosphere of marine algae have a noteworthy effect on phytoplankton growth and proliferation via nutrient exchanges and biochemical communications (1, 2). Skeletonema tropicum is a warm water diatom species restricted to tropical and subtropical areas (3, 4). Several studies have shown that *Skeletonema* spp. have a complex commensal microbiota (5, 6), and members of the family *Rhodobacteraceae* have been frequently found to be associated with *Skeletonema* and other diatoms in long-term cultures (5, 7). The chain-forming *Skeletonema* aggregates serve as complex phycosphere systems to study algal-epibiont interactions (8, 9), while the limited genomic information available about the commensal microbiota hampers our understanding of microbial roles in these microenvironments (10, 11).

Here, we report the circular genome sequence of Cognatishimia activa (“*Thalassobius activus*”) SOCE 004, which was assembled from the microbiome of the diatom *S. tropicum*. The *S. tropicum* strain was obtained from the Research Center for Harmful Algal and Marine Biology of Jinan University; the strain was originally collected from the Pearl River Estuary (113.71°E, 21.96°N). Since its isolation, it has been continuously cultivated in artificial seawater supplemented with f/2 medium in a laboratory for more than 8 years. Cultures have been maintained at 20°C under a 12:12-h light/dark regime with a light intensity of 100 μmol photons m^−2^·s^−1^. This nonaxenic culture provides a great opportunity to identify long-term bacterium-diatom interactions.

DNA was extracted using the Biospin bacteria genomic DNA extraction kit (Bioer Technology, Hangzhou, China), following the instructions of the manufacturer. Briefly, cells growing in the logarithmic phase were lysed with proteinase K and sodium dodecyl sulfate. Then, genomic DNA was extracted using the column extraction method and quantified using a NanoDrop 2000 spectrophotometer (Thermo Fisher Scientific, MA, USA). Finally, the DNA was sequenced using a combination of the QitanTech Nanopore (Qitan Technology Co., Beijing, China) and Illumina (Biomarker Technology, Beijing, China) sequencing platforms. For QitanTech Nanopore sequencing, a library was prepared using the QSK v1.1.1 kit according to the manufacturer’s instructions (Qitan Technology Co.) and sequenced on a QCell-384 flow cell. This Nanopore sequencing generated 4.6 Gbp raw data (quality, >Q7; 1.76 million reads), with an *N*_50_ value of ~6.24 Kbp and a mean read length of ~2.62 Kbp. For Illumina sequencing, a paired-end short-read (2 × 150-bp) library was prepared following the Illumina Nextera XT protocol and sequenced on an Illumina NovaSeq 6000 platform (Biomarker Technology, Beijing, China). The Illumina sequencing resulted in 39.74 Gbp clean reads (quality, >Q30; 132 million reads).

**FIG 1:**
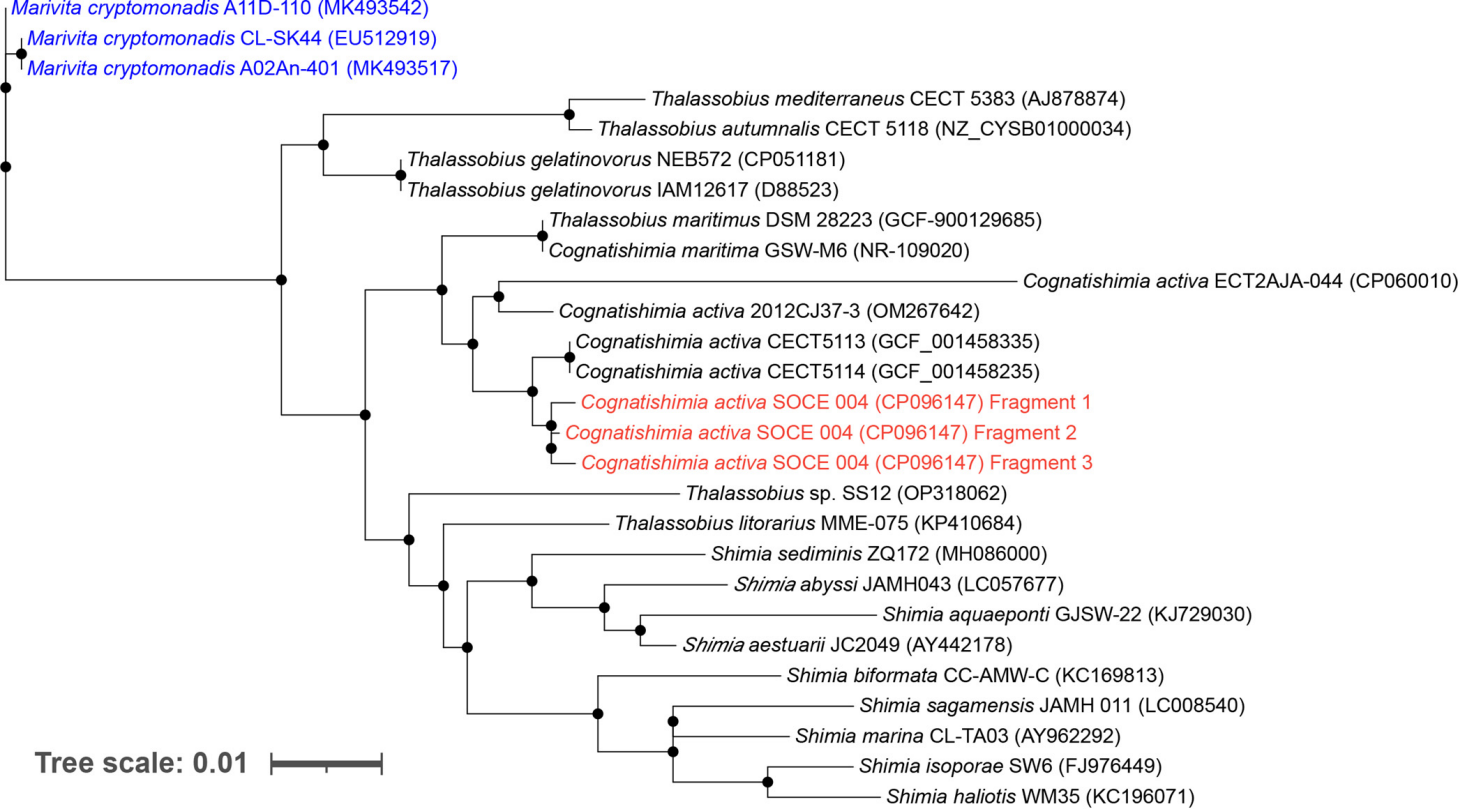
Maximum likelihood phylogenetic tree of *C*. *activa* and other closely related species. Closely related strains were obtained from the NCBI database, and only 16S rRNA sequences with valid names were selected. The 16S sequences were aligned using MUSCLE 5.1. The maximum likelihood phylogenetic tree was inferred from sequence alignment of the best-fitting model automatically selected using ModelFinder in IQ-Tree v1.6.12. The tree file generated was visualized using the iTOL Web server. The lengths of the branches represent phylogenetic distances from the reference genome. The three *C*. *activa* 16S sequences are shown in red. Three *Marivita* strains (highlighted in blue) were used as the outgroup to root the tree.

The Nanopore reads were base called and demultiplexed using Hound v1.1 (Qitan Technology Co.). Adapter sequences were removed using Porechop v0.2.4 (12). The sequence quality was assessed using NanoPack v1.25.0 (13). Flye v2.9 (14) and Canu v2.2 (15) were used to assemble the long-read sequences generated by the QitanTech Nanopore sequencing. The assemblies were then polished using Pilon v1.24 (16) and NextPolish v1.4.0 (17) with short reads generated using the Illumina platform. Unicycler v0.4.8 (12) was used to identify and clean circular contigs from the polished assemblies. A circular contig with a 16S rRNA gene similar to that of *C*. *activa* strain 2012CJ37-3 (98.65% identity via BLASTN) was selected for further analysis. A maximum likelihood phylogenetic tree was constructed using IQ-Tree v1.6.12 and visualized using the iTOL Web server (https://itol.embl.de) (Fig. 1). All tools were run with default parameters unless otherwise specified.

The circular assembly of *C. activa* SOCE 004 was annotated using the NCBI Prokaryotic Genome Annotation Pipeline (PGAP) (18). The complete genome sequence is 3,211,994 bp long, with an average G+C content of 53.69%. In total, 3,195 genes were annotated in the *C. activa* SOCE 004 genome. Among them, 3,133 are coding sequences (CDSs), 50 are tRNAs, 9 are rRNAs (5S, 16S, and 23S), and 3 are noncoding RNAs (ncRNAs). Several genes involved in nitrogen metabolism were also predicted, including nitrite/sulfite reductase (*nirD* and *nirB*) and denitrifying reductase (*narI*, *narH*, and *narJ*) genes. The availability of this genome will help us understand how *Cognatishimia* spp. adapt to the *Skeletonema* phycosphere.

### Data availability.

The complete genome sequence of *C. activa* SOCE 004 has been deposited at GenBank under the accession number CP096147. All data are available under BioProject accession number PRJNA827970 and BioSample accession number SAMN27646840. The raw Illumina and Nanopore data were submitted to the SRA and are available via accession numbers SRR22269038 and SRR22262228, respectively.
